# Health and Health Care of Mothers and Children in a Suburban Area of Luanda, Angola

**DOI:** 10.1007/s10900-013-9808-4

**Published:** 2013-12-27

**Authors:** João Baptista Humbwavali, Camila Giugliani, Bruce Bartholow Duncan, Erno Harzheim, Antônio Carlile Holanda Lavor, Míria Campos Lavor, Maria Idalice Barbosa, Patrícia Barros Thomas, Lisiane Hauser

**Affiliations:** 1 Postgraduate Program of Epidemiology, Federal University of Rio Grande do Sul (UFRGS), Porto Alegre, Brazil; 2 Department of Social Medicine, Federal University of Rio Grande do Sul (UFRGS), Rua Ramiro Barcelos, 2400/2° andar, CEP 90035-003 Porto Alegre, RS Brazil; 3 Oswaldo Cruz Foundation (FIOCRUZ), Fortaleza, Ceará Brazil; 4 Fortaleza, Ceará Brazil; 5 Post-graduate Program of Collective Health, Federal University of Ceará (UFC), Fortaleza, Brazil; 6 Brazilian National School of Public Health (ENSP), Rio de Janeiro, Brazil; 7 Institute of Health Sciences, Agostinho Neto University (UAN), Luanda, Angola; 8 Institute of Health Technology Assessment (IATS), Porto Alegre, Brazil

**Keywords:** Primary health care, Maternal health, Child health, Community health planning, Angola

## Abstract

Population health data available in Angola are often insufficient to guide the planning of health interventions. To address this gap, the goal of the present study was to investigate the health of mothers and infants in a suburban municipality in Luanda (Cacuaco), in order to provide a baseline for future comparisons. This was a prevalence study investigating infants younger than 2 years of age and their mothers. Mothers were interviewed, and children’s height and weight were measured. Of 749 mothers interviewed, 98.5 % (95 % CI 98.2–99.1 %) had at least one prenatal visit and 51.7 % (95 % CI 47.4–56.3 %) had a health card. Most mothers with a health card had their first prenatal visit before the 20th week of pregnancy, and had at least four prenatal visits; 81.1 % (95 % CI 78.3–84.1 %) of mothers also had their child’s health card. Prevalence of exclusive breastfeeding at 6 months was 19 % (95 % CI 16.2–23.1 %). Prevalence of low height-for-age and low BMI-for-age were 32 and 6 %, respectively. Mothers with higher education levels were more likely to have had their first prenatal visit earlier, to have had more prenatal visits, to have given birth at a health facility, and to have her own and her child’s health cards. Results showed a high prevalence of prenatal care and a low frequency of acute malnutrition. Maternal education level, among factors studied, was the predominant correlate of more positive health behaviors. These findings suggest important progress of mother and child health in Cacuaco, and may serve as a baseline for the planning of health interventions.

## Introduction

The health situation in Angola is in an alarming state, as the country is still reeling from the effects of a 30-year long civil war, which ended as recently as 2002. Life expectancy at birth in Angola is estimated to be 52 years, and the mortality rate in children younger than 5 years is 161/1,000 [1]. The most common causes of child mortality in Angola today are preventable diseases such as malaria and respiratory infections [1].

In an effort to restructure the country’s health system, the Health Ministry of Angola has recently implemented a program to reform municipal health systems in the country, with the aim of reducing mother and child mortality [2]. This program has focused mainly on strengthening Primary Health Care (PHC), and involved, for instance, the implementation of the Community Health Agents Program (PACS) in 2007.

A PHC-based health system is composed of structural and functional elements that guarantee universal coverage and access to health services, provide comprehensive care and engage families and communities in the planning and implementation of health interventions [3]. Recently, a World Health Report entitled “Primary health care: now more than ever” [4] revived the principles of the Alma-Ata Declaration [5], and highlighted the importance of investing heavily in PHC networks.

The successful planning and implementation of PHC interventions depend strongly on the availability of data on the health situation in the country. The quality assessment of health facilities, and the monitoring of the progress and results of health interventions are also important to ensure the efficacy of these measures and to tailor them to the target population. Given that only crude estimates exist of health parameters in Angola, field studies and registries are required to provide accurate and representative data on health care use in the country. A literature search retrieved no studies on health and health care in Luanda and its surrounding area. Such studies could provide a baseline from which to assess the effect of health care policy and interventions on the health situation of the Angolan population. To address this gap, the present study aimed to describe the health situation of mothers and infants under 2 years of age in a municipality in Luanda so as to (1) determine the demographic profile of this population and their patterns of health facility use; and (2) obtain a baseline for future comparisons of the health situation in the country. The epidemiological data obtained in the present study could also contribute to the planning of health interventions and PHC initiatives.

## Methods

### Study Location

The study was conducted in Cacuaco, one of the seven municipalities of Luanda, the capital of Angola. The population of Cacuaco is estimated at 700,000, and the city is divided into three administrative regions: Cacuaco Sede (137,000 inhabitants), Kikolo (480,000 inhabitants) and Funda (81,000 inhabitants). A reference public health service was located in Cacuaco Sede, a smaller health center was located in Kikolo, and a number of private health centers and units administrated by non-governmental organizations were also found in the districts studied.

### Study Design, Period and Population

This prevalence study was conducted on children younger than 2 years as well as on their mothers. Data was collected from August 1st to September 26th, 2010.

### Sample

A sample of 700 children enables the estimation of the prevalence of the main outcomes studied, assuming a variation of 10–40 % (corresponding to the prevalence of low BMI-for-age and low height-for-age, respectively), with 5 % precision, and considering a cluster design effect of 1.5.

### Inclusion Criteria

All children younger than 2 years (from 0 to 1 year, 11 months and 29 days) who lived in the study area were eligible for the study; those whose mothers had lived in the district for less than a year or did not live with the child were excluded. If more than one child younger than 2 years lived in the same household, the older child was included in the study. In the case of twins, only the firstborn was included. Participant loss was considered when the mother was absent on at least three interviewer visits to the household, and refusals to participate were considered when mothers did not agree to take part in the study.

### Site Selection and Data Collection

Participants were recruited from four districts, of which two were in Kikolo (Boa Esperança and Balumuca) and two in Cacuaco Sede (Bate Chapa and Forno do Sal). Districts were selected based on the following criteria: availability of neighborhood maps, authorization by resident committees and safety for researchers walking around the area. To facilitate data collection, the districts were divided into microareas with approximately 100 families each. The first microarea to be sampled in each district was randomly selected, and data was then collected in areas surrounding the initial location, until the target sample size was attained. One household in each microarea was randomly selected as a starting point for the survey, and every third house to the right of the index house was then visited by the interviewers, who consisted of a team of 16 Angolan individuals who completed a 5-day intensive training course prior to the study. The training involved simulated interviews and pilot questionnaire. After training was completed, four teams were assembled, each of which had one field coordinator, four interviewers and one area supervisor. Two representatives from the local health department also provided support to the research team.

The coordinators were responsible for identifying eligible children, and the interviewers for administering a structured questionnaire to the children’s mothers. Coordinators also collected data from the children’s health cards and the mother’s pregnancy card, and measured children’s height and weight using stadiometers and digital scales. All questionnaires were reviewed and coded by field coordinators on the same day the interviews were conducted.

### Variables

Demographic, socioeconomic, and health data were collected from mothers and children, as was information regarding patterns of health facility use. Data regarding prenatal care, immunization and children’s weight monitoring were obtained from the mother’s information, as well as from the records in the mother’s pregnancy card and the child’s health card. However, only the health card information was used in data analysis, as these data were more reliable. The presence of hypochlorite and mosquito bednets in the homes was also assessed by the interviewers.

The Kessner Index [6] modified by Takeda [7] was used to measure the quality of prenatal care. According to these criteria, prenatal care is considered adequate when initiated before the 20th week of gestation, and when the mother had at least six health care visits throughout the pregnancy. Post-natal care was considered adequate when it was obtained within 7 days of childbirth.

Immunization was assessed based on local vaccination calendars. Vaccines were considered delayed when they were not recorded on the health cards, or when the children were over 1 month older than the recommended age for receiving a given vaccine. The same 1 month delay was considered for adequacy of child’s weight monitoring. The growth curves on the child’s health card were used as reference, where weight was expected to be recorded at birth, and at 2, 4, 6, 9, 12 and 18 months of age.

Exclusive breastfeeding was assessed in children between 0 and 6 months, and breastfeeding at 12 months was assessed in children aged from 12 to 15.9 months [8, 9].

Socioeconomic status was scored according to the system proposed by Krefis et al. [10]. The variables included in the socioeconomic status index were: type of material from which the house was built, access to piped water, electricity, and the presence of a bathroom inside the house. Families received a score ranging from 0 to 10, where scores below five were indicative of lower socioeconomic status, and scores between six and ten points suggested higher socioeconomic status. Parental educational level was ascertained as the grade year of schooling attained.

### Data Analysis

Questionnaires were coded, scanned and entered into a database using the Teleform^®^ software package. Frequencies were obtained using the SPSS software, version 18.0, and prevalence ratios were calculated using the Stata 9 software. The association between socioeconomic status, maternal education level and health outcomes (having a health card, time of the first prenatal visit, number of visits throughout the pregnancy, all children being alive, interval between childbirth and child immunization) was investigated using Pearson’s Chi square test, with significance considered at *p* < 0.05, and controlling for the cluster design effect.

### Ethical Considerations

The present study was conducted as part of the project entitled *Developing PHC services in Angola: a proposal for the assessment of PACS in Luanda*, which was approved by the Research Ethics Committee of the Federal University of Rio Grande do Sul. Data collection was also authorized by the Luanda Province Health Department. All participating mothers signed an informed consent document prior to being interviewed for the present study.

## Results

A total of 1,360 homes were visited in 49 microareas of the four selected districts. A total of 749 children were included, as were their mothers. A total of 42 (5.7 %) families were excluded, 111 (15.0 %) were lost and 10 (1.4 %) refused to participate.

Table 1 displays the socio demographic and economic characteristics of participants, as well as data regarding their living conditions. Mean maternal age was 26 years, and mean child age was 10.6 months. A total of 85.3 % of the mothers interviewed were married or lived with their partner, and 42.5 % had up to 4 years of education. The most commonly reported occupations were housewife (42.4 %) and self-employed (43.9 %), with most individuals in the latter category working as salespeople.

**Table 1:** Socio demographic and economic characteristics of mothers and infants under 2 years in the Cacuaco municipality, 2010 (N = 749)

Variable^a^	N (%) or mean	95 % CI or SD
Mother’s age (years)	26.1	±6.2
Child’s age (months)	10.6	±6.9
Child age range (months)		
0–6	269 (36.0)	32.5–36.9 %
7–12	152 (20.3)	17.6–23.3 %
13–18	200 (26.7)	23.7–29.7 %
19–24	127 (17.1)	14.3–19.8 %
Mother’s marital status		
Married/common law	639 (85.3)	82.6–87.9 %
Separated/divorced	32 (4.3)	3.1–5.7 %
Widowed	2 (0.3)	0.0–0.7 %
Mother’s education level		
0–4 years	317 (42.5)	38.9–45.8 %
5–8 years	310 (41.6)	37.9–45.2 %
9 or more years	119 (16.0)	13.4–18.5 %
Partner’s education level		
0–4 years	34 (6.9)	4.5–9.3 %
5–8 years	212 (42.9)	38.3–7.2 %
9 or more years	248 (50.2)	46.0–54.5 %
Mother’s occupation		
Housewife	316 (42.4)	38.8–46.0 %
Self-employed	327 (43.9)	40.3–47.2 %
Public sector employee	12 (1.6)	0.8–2.6 %
Private sector employee	23 (3.1)	2.0–4.4 %
Student only	67 (9.0)	7.0–11.1 %
Partner’s occupation		
Public sector employee	165 (25.2)	22.0–28.7 %
Private sector employee	331 (50.5)	46.4–54.4 %
Self-employed	129 (19.7)	16.5–22.7 %
Student only	8 (1.2)	0.5–2.1 %
Unemployed	22 (3.4)	2.0–4.9 %
Living arrangement		
Own home	457 (61.0)	57.5–60.4 %
Rented home	261 (34.8)	31.6–38.3 %
Occupied home	31 (4.1)	2.7–5.5 %
Toilet facilities available		
Yes	728 (97.2)	95.9–98.4 %
No	21 (2.8)	1.6–4.1 %
Toilet location		
Inside the home	55 (7.6)	5.6–9.6 %
Outside the home, family use only	303 (41.6)	38.2–45.5 %
Outside the home, shared with other families	370 (50.8)	47.3–54.4 %
Source of water in the house		
Pipe borne	20 (2.7)	1.6–3.9 %
Cistern (shared)	26 (3.6)	2.2–4.9 %
River/lake/canal	1 (0.1)	0.0–0.4 %
Spring (shared)	305 (40.8)	37.3–44.3 %
Tank (shared)	385 (52.8)	49.4–56.4 %
Other	12 (1.6)	0.8–2.6 %
Drinking water treatment—mother information		
Boiled	30 (4.0)	2.7–5.6 %
Hypochlorite treatment	537 (71.7)	68.5–74.9 %
Filtered	1 (0.1)	0.0–0.4 %
No treatment	181 (24.2)	21.0–27.2 %
Presence of hypochlorite in the home—interviewer observation^b^		
Yes	259 (60.9)	56.2–66.1 %
No	166 (39.1)	33.9–43.8 %
Presence of mosquito nets in the home—interviewer observation		
Yes	383 (51.1)	46.2–56.1 %
No	366 (48.9)	44.0–54.1 %
Source of electricity		
Supply network	521 (69.5)	61.2–77.18 %
Generator	125 (16.7)	11.0–22.3 %
No electricity	103 (13.8)	10.1–18.4 %
Presence of a refrigerator in the home		
Yes	446 (59.5)	53.0–66.7 %
No	303 (40.5)	34.1–47.0 %
Presence of a television in the home		
Yes	662 (88.4)	86.0–90.5 %
No	87 (11.6)	9.5–14.0 %
Means of garbage disposal		
Collected by the city	248 (33.2)	30.0–36.7 %
Discarded outside the home	482 (64.6)	61.1–68.0 %
Discarded in yard around the home	3 (0.4)	0.0–0.9 %
Burning	11 (1.5)	0.7–2.4 %
Other	2 (0.3)	0.0–0.7 %
Family socioeconomic score (0–10)		
Higher income (6–10)	387 (51.7)	47.9–55.5 %
Lower income (0–5)	362 (48.3)	44.5–52.1 %

*N* number of subjects

^a^N may vary according to the data available for each variable

^b^N refers to the occasions in which it was possible to investigate the presence of hypochlorite in the home at the time of the interview

The results showed that 92.1 % of the families surveyed obtained water from collective wells or springs. During the interviews, 71.7 % of mothers reported to treating drinking water with hypochlorite, although the interviewers only found this product in 60.9 % of households. A total of 48.3 % of the families surveyed were classified as having lower socioeconomic status.

Table 2 displays the mothers’ health information. Almost all mothers (98.5 %) reported to having had at least one prenatal care visit, and in 95 % of cases, these visits occurred in public facilities. Despite the high frequency of prenatal care, only 51.7 % of mothers had a pregnancy health card. Considering the card data only, 60.4 % of mothers had four or more prenatal visits, and 76.5 % initiated prenatal care before the 20th week of gestation. However, according to the Kessner index, only 16.9 % of mothers had adequate prenatal care. A total of 69.5 % of mothers gave birth in health care facilities, while the remaining mothers reported to having given birth at home. Only 3.6 % of mothers had postnatal visits recorded on their health cards.

**Table 2:** Health data for mothers living in the Cacuaco municipality 2010 (N = 749)

Variable^a^	N(%) or mean	95 % CI or SD
Number of children per mother	3.6	±2.4
Number of live children	3.1	±1.9
Number of dead children	0.5	±0.9
Number of stillbirths	0.1	±0.3
Interval between births (months)	39.1	±23.5
History of abortion		
Yes	148 (19.8)	17.0–22.7 %
No	601 (80.2)	77.3–83.0 %
Number of abortions per mother	0.3	±0.7
At least one prenatal visit (mother information)		
Yes	720 (98.5)	97.6–99.3 %
No	11 (1.5)	1.0–2.3 %
Prenatal care in public health service	702 (96)	94.7–97.4 %
At least four prenatal visits—pregnancy health card	223 (60.4)	55.3–65.6 %
At least six prenatal visits (mother information)	71 (19.2)	15.7–23.3 %
First prenatal visit before 20th week of pregnancy	257 (76.5)	70.1–80.4 %
Adequate prenatal care—Kessner index	62 (16.9)	13.1–21.0 %
Pregnancy health card ownership—verified by interviewer		
Yes	373 (51.7)	47.1–56.4 %
No	349 (48.3)	44.2–53.1 %
Received tetanus vaccine during pregnancy (according to the mother’s health card)		
Yes. at least one dose	350 (96.7)	95.1–99.3 %
Not necessary. As the mother had already received the vaccine	1 (0.3)	0.0–0.4 %
Not registered on the health card	11 (3.0)	1.0–5.2 %
Malaria prevention treatment^b^ (according to the mother’s health card)		
Yes (one dose)	101 (28.1)	22.3–34.2 %
Yes (two or more doses)	247 (68.6)	62.1–75.8 %
Not registered on the health card	12 (3.3)	1.0––5.4 %
Supplementation with iron and/or folic acid (according to the mother’s health card)		
Yes	367 (99.2)	98.0–100 %
Not registered on the health card	3 (0.8)	0.0–2.1 %
Postnatal visit (according to the mother’s health card)		
Yes	9 (3.6)	1.1–6.3 %
No	241 (96.4)	94.1–99.5 %
Type of delivery (mother information)		
Spontaneous	711 (94.9)	93.2–95.3 %
Cesarean	38 (5.1 %)	4.1–7.3 %
Place of delivery (mother information)		
Health facility	520 (69.5)	64.1–75.3 %
At home	228 (30.5)	25.2–36.5 %

^a^N may vary according to the data available for each variable

^b^Preventive Intermittent Treatment: recommended to all pregnant women in the country. Consists of at least two doses of anti-malaria drugs (in Angola, involved treatment with 500 mg sulfadoxine and 25 mg pyrimethamine at the time of data collection)

Results showed that 43 % of mothers had fallen ill at some point during the pregnancy. The most frequently reported illnesses were malaria (n = 204; 27 %; 95 % CI 24.1–31.4 %) and high blood pressure (n = 94; 13 %; 95 % CI 10.2–15.6 %). Inquiries into payment for public health services were also made, and 263 (35 %; 95 % CI 31.0–40.3 %) mothers were found to have paid for prenatal care, medical tests or medication, and 268 (36 %; 95 % CI 31.2–40.0 %) reported having paid for childbirth. As for children’s visits to public health facilities, 205 (27.4 %; 95 % CI 22.1–33.0 %) mothers reported to having paid for these and 111 (14.8 %; 95 % CI 11.2–18.6 %) paid for medication supplied by the health service itself.

Table 3 displays the health data for children included in the present study. Mothers had the health cards for 81.1 % of children in the sample. Only 19 % of the children were exclusively breastfed until 6 months of age and 88.6 % were still being breastfed at 12 months. A total of 74 and 71.2 % of children were up to date for their age for pentavalent vaccine doses and weight monitoring, respectively. The survey also showed that 36 % of children had low to very low height-for-age. The prevalence of low BMI-for-age (Z scores below −2) was of 6 %. Two percent of children were found to be obese, 3.9 % were overweight and 17.2 % were at risk for overweight.

**Table 3:** Health data for children living in the Cacuaco municipality. 2010 (N = 749)

Variable^a^	N (%) or mean	95 % CI or SD
Prevalence of exclusive breastfeeding at 6 months	51 (19)	16.2–23.1 %
Prevalence of breastfeeding at 12 months^b^	90 (73)	65.3–81.0 %
Ownership of child health card (verified by interviewer)		
Yes	602 (81.1)	78.3–84.1 %
No	140 (18.9)	16.1–22.3 %
Birth weight (g) (according to the child’s health card)	3,262.4	± 518.7
<2,500 g	39 (8.5)	5.9–10.9 %
2,500–4,000 g	395 (86.4)	83.4–89.7 %
>4,000 g	23 (5.0)	3.1–7.0 %
BCG vaccine^c^		
Up to date	570 (96.3)	94.6–97.6 %
Delayed	22 (3.7)	2.4–5.4 %
Poliomyelitis vaccine—3rd dose^c^		
Up to date	198 (75.9)	70.2–82.1 %
Delayed	63 (24.1)	19.0–30.2 %
Pentavalent vaccine 3rd dose^c^		
Up to date	199 (74.0)	68.1–80.3 %
Delayed	70 (26.0)	20.0–32.1 %
Mumps vaccine^c^		
Up to date	141 (67.5)	60.3–75.2 %
Delayed	68 (32.5)	25.1–41.0 %
Yellow fever vaccine^c^		
Up to date	111 (60.7)	53.1–69.8 %
Delayed	72 (39.3)	31.2–47.1 %
Weight registers (according to the child’s health card)		
Up to date	426 (71.2)	68.1–75.2 %
Delayed	172 (28.8)	25.4–32.1 %
Height-for-age^d^		
Z score < −3.0 (very low)	96 (13.2)	10.8–15.7 %
Z score between −3.0 and −2.01 (low)	136 (18.8)	15.7–21.7 %
Z score between −2.0 and −1.01 (low to normal)	194 (26.8)	23.4–30.2 %
Z score between −1.0 and 1.0 (normal)	243 (33.5)	30.3–37.0 %
Z score between 1.01 and 2.0 (normal to high)	29 (4.0)	2.5–5.5 %
Z score between 2.01 and 3.0 (high)	13 (1.8)	0.8–2.8 %
Z score > 3.0 (very high)	14 (1.9)	1.0–2.9 %
BMI-for-age^d^		
Z score < −3.0 (very thin)	15 (2.1)	1.1–3.2 %
Z score between −3.0 and −2.01 (thin)	28 (3.9)	2.6–5.4 %
Z score between −2.0 and −1.01 (thin to normal)	98 (13.7)	11.2–16.2 %
Z score between −1.0 and 1.0 (normal)	411 (57.3)	53.6–60.8 %
Z score between 1.01 and 2.0 (at risk for overweight)	123 (17.2)	14.5–19.9 %
Z score between 2.01 and 3.0 (overweight)	28 (3.9)	2.6–5.3 %
Z score >3.0 (obese)	14 (2.0)	1.0–3.1 %

*N* Number of subjects, 9*5* *% CI* 95 % confidence interval, *SD* standard deviation, *g* grams, *BMI* body mass index

^a^N may vary according to the data available for each variable. ^b^ Prevalence in children aged between 12 and 15.9 months of age. ^c^ Children who were not old enough to be immunized were not considered part of the target population. ^d^ According to anthropometric data collected on the day of the interview

According to the mothers’ reports, 262 children (35 %; 95 % CI 31.2–40.0 %) had had an episode of diarrhea in the 15 days preceding the interview, 247 (33 %; 95 % CI 30.1–36.3 %) had had a fever and 306 (41 %; 95 % CI 36.2–46.4 %) had had a cough. A total of 273 (36.5 %; 95 % CI 33.3–40.2 %) children had been taken to health facilities due to illness in the previous 3 months and 28 children (3.7 %; 95 % CI 2.0–5.2 %) had been admitted to hospital in the past 12 months [18 due to malaria (64 %), seven due to diarrhea (25 %) and four for respiratory infections (14 %)].

Only 200 (27 %; 95 % CI 23.2–31.3 %) children had a birth certificate at the time of the study. The main reason children had not been registered, according to mothers, was the unavailability of parents’ identification documents (n = 270; 63.5 %; 95 % CI 57.3–70.1 %).

Table 4 displays the association between predictor variables and some of the health outcomes analyzed in the present study. Mothers with a higher level of education (more than 9 years), as opposed to those with less education (zero to 4 years) were 32 % more likely to have a pregnancy card, 18 % more likely to have their child’s health card, 18 % more likely to have started prenatal care before 20 weeks of gestation, 79 % more likely to have had four or more prenatal appointments, 80 % more likely to have given birth at a health facility, and 76 % more likely to have all of their children currently alive (*p* < 0.05 for all). Those with higher socioeconomic status were 26 % more likely to have had four or more prenatal visits and 13 % more likely to have given birth at a health facility.

**Table 4:** Prevalence of selected health and health care indicators according to maternal education level and socioeconomic status (N = 749)

Predictor variable	Maternal education level					Socioeconomic status				
Outcome (n)	0–4 years n (%)	0–8 years n (%)	≥9 years n (%)	Total n (%)	PR (95 % CI) *P* value	n for outcome	Low n (%)	High n (%)	Total n (%)	PR (95 % CI) *P* value
Pregnancy health card ownership (n = 531)										
Yes	129 (62.0)	167 (72.6)	76 (81.7)	372 (70.1)	1.32 (1.14–1.51)	n = 532	171 (67.3)	202 (72.7)	373 (70.1)	1.08 (1.0–1.4)
No	79 (38.0)	63 (27.4)	17 (18.3)	159 (29.9)	0.001		83 (32.7)	76 (27.3)	159 (29.9)	0.18
Child health card ownership (n = 739)										
Yes	261 (80.4)	264 (86.0)	112 (94.9)	637 (86.2)	1.18 (1.15–1.43)	n = 742	308 (86.0)	331 (92.2)	639 (86.1)	1.07 (0.8–1.2)
No	53 (19.6)	43 (14.0)	6 (5.1)	102 (13.8)	0.013		50 (14.0)	53 (13.8)	103 (13.9)	0.95
Prenatal care before 20th week (n = 335)										
Yes	88 (70.4)	120 (79.5)	49 (83.1)	257 (76.7)	1.18 (1.01–1.49)	n = 257	115 (75.2)	142 (77.6)	257 (76.5)	1.03 (0.8–1.4)
No	37 (29.6)	31 (20.5)	10 (16.9)	78 (23.3)	0.009		38 (24.8)	41 (22.4)	79 (23.5)	0.60
At least four prenatal visits (n = 368)										
Yes	57 (44.9)	104 (63.0)	61 (80.3)	222 (60.3)	1.79 (1.38–2.11)	n = 369	90 (52.9)	133 (66.8)	223 (60.4)	1.26 (1.1–1.7)
No	70 (55.1)	61 (37.0)	15 (19.7)	146 (39.7)	<0.001		80 (47.1)	66 (33.2)	146 (39.6)	0.007
Delivery at health facility (n = 745)										
Yes	171 (54.1)	232 (74.8)	116 (97.5)	519 (69.7)	1.80 (1.50–1.91)	n = 748	235 (65.1)	285 (73.6)	520 (69.5)	1.13 (1.1–1.4)
No	145 (45.9)	78 (25.2)	3 (2.5)	226 (30.3)	<0.001		126 (34.9)	102 (26.4)	228 (30.5)	0.011
All children currently alive (n = 744)										
Yes	169 (53.5)	250 (80.6)	111 (94.1)	530 (71.2)	1.76 (1.57–1.80)	n = 747	253 (70.1)	278 (72.0)	531 (71.1)	1.03 (0.8–1.1)
No	147 (46.5)	60 (19.4)	7 (5.9)	214 (28.8)	<0.001		108 (29.9)	108 (28.0)	216 (28.9)	0.60
≥3 years between births (n = 569)										
Yes	126 (43.8)	96 (42.9)	34 (59.6)	256 (45.0)	1.36 (1.31–1.43)	n = 572	120 (43.6)	137 (46.1)	315 (56.4)	1.06 (0.9–1.3)
No	162 (56.3)	128 (57.1)	23 (40.4)	313 (55.0)	0.06		155 (56.4)	160 (53.9)	157 (44.9)	0.55
BCG vaccine up to date for age (n = 557)										
Up to date	220 (97.3)	220 (94.8)	95 (96.0)	535 (96.1)	0.99 (0.52–1.44)	n = 559	254 (94.8)	283 (97.3)	537 (96.1)	1.03 (1.0–1.9)
Delayed	6 (2.7)	12 (5.2)	4 (4.0)	22 (3.9)	0.38		14 (5.2)	8 (2.7)	22 (3.9)	0.13
Poliomyelitis 3rd dose up to date (n = 260)										
Up to date for age	70 (75.3)	78 (73.6)	49 (80.3)	197 (75.8)	1.07 (0.75–1.24)	n = 261	82 (73.9)	116 (77.3)	198 (75.9)	1.05 (0.8–1.3)
Delayed	23 (24.7)	28 (26.4)	12 (19.7)	63 (24.2)	0.61		29 (26.1)	34 (22.7)	63 (24.1)	0.52
Pentavalent vaccine 3rd dose up to date (n = 268)										
Up to date for age	71 (71.7)	79 (73.1)	48 (78.7)	198 (73.9)	1.10 (0.80–1.25)	n = 269	84 (73.7)	115 (74.2)	199 (74.0)	1.01 (0.7–1.2)
Delayed	28 (28.3)	29 (26.9)	13 (21.3)	70 (26.1)	0.61		30 (26.3)	40 (25.8)	70 (26.0)	0.93
Mumps vaccine up to date (n = 208)										
Up to date for age	47 (61.0)	54 (66.7)	40 (80.0)	141 (67.8)	1.31 (0.90–1.34)	n = 209	61 (71.8)	80 (64.5)	141 (67.5)	0.90 (0.5–1.0)
Delayed	30 (39.0)	27 (33.3)	10 (20.0)	67 (32.2)	0.08		24 (28.2)	44 (35.5)	68 (32.5)	0.27

*PR* prevalence ratio, *95* *% CI* 95 % confidence interval

## Discussion

Considering the fact that Angola in recent years has been cited as having one of the world’s highest infant mortality rates, these results suggest rapid, though incomplete progress in terms of organizing maternal and child health services. The vast majority of mothers surveyed had at least one prenatal visit and obtained prenatal care in public health centers. A high number of women also had their first prenatal visit before the 20th week of gestation, and had had at least four prenatal visits throughout the pregnancy. However, the number of postnatal visits was low, as was the number of children with birth certificates. Most of the mothers had pregnancy cards and carried their children’s cards, and high rates of vaccination were observed. Most of the children were also up to date for their weight assessments. Malaria and diarrhea were the most commonly reported diseases in the sample. High disparity in educational attainment exists, with 42.5 % of women having only 0–4 years of education while 16 % had 9 or more years. Maternal education level, more than socioeconomic status, was found to correlate with more positive health behaviors and outcomes.

The present findings can be compared with data obtained relatively recently in other studies conducted in Angola. Studies have reported that approximately 80 % [11] of mothers in the country had one or more prenatal visits, and that 61 % of mothers in urban regions of Angola had four or more visits throughout the pregnancy [12]. The prevalence of low birth weight in the present sample was lower than that found in previous studies in the literature [11]. As for the place of delivery, other studies, while estimated that only 47 % of all births in Angola occurred at a health facility [11], found that 67.6 % of births among those living in urban areas did occur at a health facility [12], a similar percentage to that obtained in the present study. As for the causes of morbidity, the proportion of children who presented with fevers or diarrhea in the 15 days preceding the interview was higher than that reported by other sources [13]. A total of 71.7 % of the mothers who responded to the present survey reported to using hypochlorite to treat drinking water in their homes, as opposed to only 52.5 % in the IBEP survey (Integrated Survey for the Well-Being of Population) [12]. However, it is important to note that the product was only found in 60.9 % of the households visited by the interviewers in the present study. Malaria and other acute diarrheic diseases were the most frequently reported causes of disease in the present investigation, and were also reported to be the main causes of morbidity in a diagnostic study conducted in Cacuaco in 2008 [14]. Although the rates of exclusive breastfeeding until 6 months of age found in the present study were higher than WHO estimates for 2000–2009 [11], these rates should still be considered rather low.

Adverse health outcomes were more strongly associated with low maternal education than with low socioeconomic status. However, the latter variable was related to difficulty accessing health services. The IBEP survey [12] has reported that access difficulties are the main barriers to health service use (56.2 %, amongst other barriers considered). A UNICEF survey conducted in 2001 [13] found that higher levels of education were associated with a higher frequency of prenatal visits, a higher likelihood of giving birth with the assistance of skilled personnel and of owning the child’s health card, and with better immunization coverage. According to Ester et al. [15], low education levels, especially among mothers, are important predictors of child mortality. The present data show the importance of public policy changes that focus on women’s education and empowerment, as stressed by the WHO Commission for Social Determinants of Health in 2008 [16].

It is important to note that the results with which the present findings were compared [11–13] were obtained from slightly different populations (the UNICEF and IBEP surveys included children aged between 0 and 4 years) through studies with a somewhat different design. Notwithstanding, these were the most relevant studies with which to compare the present results. Previous surveys [17] in the Cacuaco municipality have shown that the high cost of maternal and child health cards, as well as of mosquito nets and hypochlorite, made it so that these items were only accessible to a much smaller part of the population than currently found. According to the UNICEF-MICS survey conducted in 2001, only 40.4 % of children in Luanda had a health card [13]. In the present study, this percentage increased to 81 %. Similarly, access to medical visits and to safe childbirth used to be mostly contingent upon the payment of fees. This practice has decreased sharply over recent years, and in the present study, the percentage of mothers who reported to have had to pay for her own or her child’s health care amounted to only 20–30 %.

The comparisons between the present findings and those of previous surveys encourage an optimistic perspective of the health situation in Angola. A study of the factors associated with high child mortality rates in 47 countries in Sub-Saharan Africa [15] found that such rates were inversely correlated with prenatal care attendance and birth assisted by a skilled professional. The present study obtained promising results regarding prenatal health care in Angola. However, according to the Kessner index, the prevalence of adequate prenatal care was low, probably due to its six visit cut-off. Although an increasing number of women have been giving birth in health care facilities, there is still much room for improvement on this front. The present findings suggested that more human and financial resources must be invested in improving access to maternal and child health services in Angola. According to Ester et al. [15] sewage treatment is another important factor related to child mortality, outlining a fundamental intervention in the context of Cacuaco.

The present data, when matched with the literature, suggest that a strong emphasis should be placed on community health workers (CHW) in Angolan PHC policies and planning. Evidence of the effectiveness of CHW interventions in other regions with similar epidemiological characteristics and needs suggests that the implementation of such initiatives in Angola could have a positive impact [18, 19]. A recent systematic review [18] conducted in Brazil, for instance, found that, out of all outcomes studied, mother–child health showed the largest improvements from CHW interventions. In Rwanda, a country whose post-war social structure is similar to that of Angola, the government has invested in CHW programs to improve mother–child health care, and results are already beginning to show: there have been significant increases in immunization rates and in the use of insecticide-treated mosquito nets. Childhood mortality has significantly decreased [20].

It is important to note that this study, as it provides detailed data in underprivileged communities in Luanda, could serve as a baseline for future studies and surveys. Studies such as the present one could make significant contributions to the assessment of the effectiveness of different health interventions [21].

The present results must be interpreted in light of a few limitations. The fact that variables were collected from maternal and child health cards rather than from the mother’s information (which was assumed to be less reliable) produced a reasonably large non-response rate, and may have led to an overestimation of the prevalence of certain desirable health behaviors.

Although much progress is still to be made in terms of mother and child health in Angola, the present results showed that health reform initiatives in the country, with improved access to health care, implementation of mobile health teams, reduced distances to reach a health center, reduced need to pay for health care, and the incipient presence of CHWs have produced promising results in the health sector. However, though the present study showed some improvement comparing to previous surveys, some important barriers to access to health care and to quality health services still exist in Angola.

In conclusion, the present findings showed promising results in the Cacuaco municipality, and may serve as a basis for the planning of interventions and as a baseline for future comparisons.
